# Is gender-based violence a confluence of culture? Empirical evidence from social media

**DOI:** 10.7717/peerj-cs.1051

**Published:** 2022-07-29

**Authors:** Rimjhim, Sourav Dandapat

**Affiliations:** Department of Computer Science and Engineering, Indian Institute of Technology Patna, Patna, India

**Keywords:** Computational social science, Social computing, Ethnography, Gender based violence, Twitter, Correlation, Hofstede Cultural Dimension, Culture, Sexual violence, Physical violence

## Abstract

Gender-based violence (GBV) has been plaguing our society for long back. The severity of GBV has spurred research around understanding the causes and factors leading to GBV. Understanding factors and causes leading to GBV is helpful in planning and executing efficient policies to curb GBV. Past researches have claimed a country’s culture to be one of the driving reasons behind GBV. The culture of a country consists of cultural norms, societal rules, gender-based stereotypes, and social taboos which provoke GBV. These claims are supported by theoretical or small-scale survey-based research that suffers from under-representation and biases. With the advent of social media and, more importantly, location-tagged social media, huge ethnographic data are available, creating a platform for many sociological research. In this article, we also utilize huge social media data to verify the claim of confluence between GBV and the culture of a country. We first curate GBV content from different countries by collecting a large amount of data from Twitter. In order to explore the relationship between a country’s culture and GBV content, we performed correlation analyses between a country’s culture and its GBV content. The correlation results are further re-validated using graph-based methods. Through the findings of this research, we observed that countries with similar cultures also show similarity in GBV content, thus reconfirming the relationship between GBV and culture.

## Introduction

Gender-based violence (GBV) is one of the most heinous and age-old violations of human rights (https://www.who.int/news-room/fact-sheets/detail/violence-against-women). GBV is evident across all parts of the globe (https://www.undp.org/content/undp/en/home/blog/2018/violence-against-women-cause-consequence-inequality.html), and it has been plaguing our society for a long back. The condition is so severe that one in three women is reported to have faced GBV (https://www.who.int/news/item/09-03-2021-devastatingly-pervasive-1-in-3-women-globally-experience-violence). With alarming instances of GBV around the world, social and governmental organisations are taking rigorous preventive measures. The quest to deliver effective preventive measures has triggered research to understand the causes and factors of GBV to provide effective preventive measures. Research in this field have found that cultural norms which comprise of societal stigma, gender-based rules, and societal prejudices are major factors that contribute to GBV (Jewkes, Jama-Shai & Sikweyiya, 2017; Elischberger et al., 2018; Raj & Silverman, 2002; Jewkes, Levin & Penn-Kekana, 2002; Bishwajit, Sarker & Yaya, 2016). GBV is pervasive across all social, economic, and national strata (Dartnall & Jewkes, 2013), but the type of GBV, the intensity of GBV, people’s reactions, and opinions for any GBV event is not the same across the globe. For example, acid attacks are a form of revenge in developing countries arising because of refusal of a marriage proposal or a love proposal, or land disputes (Bahl & Syed, 2003). However, in South America, the same acid attack results from poor relationships and domestic intolerance toward women (Guerrero, 2013). The context of GBV changes with the country, and this change is known to be an outcome of persisting culture in a country (Abrahams et al., 2014; Fulu & Miedema, 2015; Alesina, Brioschi & La Ferrara, 2016; Perrin et al., 2019; Stubbs-Richardson, Rader & Cosby, 2018). The World Health Organization (WHO) has studied cultural norms of many countries leading to various forms of GBV (https://apps.who.int/iris/bitstream/handle/10665/77936/9789241500845_eng.pdf?sequence=1isAllowed=y). The global organization World Bank also pronounced to work on such cultural and social norms to curb GBV (https://thedocs.worldbank.org/en/doc/656271571686555789-0090022019/original/ShiftingCulturalNormstoAddressGBV.pdf).

However, these researches claiming cultural norms as a driving factor behind GBV are based on cognitive studies which require significant intervention from social and cultural experts. The claims presented in these works are based upon long-term manual discerning of GBV events occurring in countries of different cultures. These researches are dependent upon survey/questionnaire-based data which can be collected only in a limited amount and can also suffer from several biases. Thus, past research lacks a large-scale, data-driven empirical research to verify the confluences between culture and GBV.

In this article, we take a step to answer the research question ”Is gender-based violence a confluence of culture?” by experimenting with large-scale social network data. The use of social network data for research around GBV is a non-conventional way to dive into the finer details of GBV. Our research analyses GBV from the lens of culture. This research is useful for social workers, policy-makers, governments, and other organizations working for the welfare of women and society (Kim, 2021). Additionally, the findings of this research can help in planning more efficient and targeted GBV policies and awareness campaigns. Social network data has already become a substitute for survey data for numerous applications. Recently, social network data has also gained much utility for research related to GBV (Hansson, Sveningsson & Ganetz, 2021; Liu et al., 2019; Chowdhury et al., 2019; Hassan et al., 2020). Online content contains a rich spectrum of information pertaining to user opinions/reactions, ongoing news/events (Blake et al., 2021), and many more (Nikolov et al., 2015; Pal et al., 2018). Thus, online content is not only a mere content but a real-time proxy for user behaviour. For this research, we consider online content related to GBV from different countries as a representative of user reactions and perspectives towards GBV. We design experiments to check for the content similarity between countries with similar cultures. Towards this goal, we perform the correlation analysis between content distance and cultural distance between countries. Further, to validate results from the correlation analysis, we also performed graph analyses. In graph analyses, we create graphs with countries as nodes and different types of distances (content distance and cultural distance) between countries is used for building edges. These graphs are compared using various graph comparison metrics.

On experimentation with Twitter content from different countries, we find a statistically significant positive correlation between GBV content distance and cultural distance. We also observed a higher similarity between the GBV content graph and the culture graph. Thus, through the findings of this research, we observe that the countries which are similar in culture also show higher similarity in GBV content. This observation is consistent with correlation analyses and graph analyses. From this observation, we can conclude that there are traces of culture in GBV content which justifies the claim of confluences of culture on GBV. The contributions of the current research can be summarized as follows:

• In this research, we explore evidence of confluence between GBV and the culture by means of an empirical study conducted over a large dataset created naturally over a long period of time on social media.
• The results obtained from this research justify the hypothesis that GBV is a confluence of culture. This hypothesis has not been tested in past literature using uncensored and unbiased social media data.
• All the experiments conducted in this research are extended to different categories of GBV and generic online content. Further, all the six dimensions of culture are also investigated. Thus, we provide a holistic analysis.
• The findings in this research are supported by correlation analyses as well as graph-based analyses. Thus, making our claims more robust.

The rest of the article is organized as follows. ‘Related Works’ details relevant past literature related to this research. ‘Dataset and Processing’ describes the collected data. ‘Methodology’ elaborates on the methodology of our experiments, and ‘Results’ shows all the results and analyses. ‘Discussions’ discusses the implications and limitations of the work. Finally, ‘Conclusion and Future Work’ concludes our work with possible future works.

## Related Works

This research is based upon three broad areas of related works i. The relation between GBV and culture ii. Social Media Content as a source of Data and iii. GBV through social media.

### GBV and culture

GBV is a social ill evident across all the countries irrespective of their economy, language, and demography. However, with country, the type of GBV, its intensity, and the reaction of people vary (Fakunmoju, Bammeke et al., 2017). For example, in the USA, dating violence is more common than in Africa where there are comparatively lesser instances of dating violence (Johnson et al., 2015). On the other hand, in Africa, intimate partner violence is more prominent as compared to North America (https://apps.who.int/iris/bitstream/handle/10665/85239/9789241564625_eng.pdf). This implies that the same GBV is represented differently in a different country. This implies that GBV is a global evil but the context of GBV changes with the country. There have been many research to understand the causes and factors leading to GBV (Jewkes, Levin & Penn-Kekana, 2002; Jewkes, Jama-Shai & Sikweyiya, 2017; Marine & Lewis, 2020). These works have claimed that a country’s culture can characterize the persisting GBV in the country. Every culture has norms, prejudices, and societal rules that design the behaviour of people towards GBV. For example, in Malawi, the concept of polygamy and dowry is evident in the culture, and these perpetuate GBV in Malawi (Bisika, 2008). Similar research in many other countries like UK (Aldridge, 2021), Ethiopia (Le Mat et al., 2019), Cambodia  (Palmer & Williams, 2017), and many other countries (Djamba & Kimuna, 2015; Raj & Silverman, 2002) have highlighted cultural norms which lead to one or other form of GBV. Not only in research but global organisations like WHO (https://www.who.int/violence_injury_prevention/violence/norms.pdf), World Bank (https://thedocs.worldbank.org/en/doc/656271571686555789-0090022019/original/ShiftingCulturalNormstoAddressGBV.pdf) have also highlighted the cultural norms of many countries that influence GBV. The socio-cultural impact is so intense that people even justify instances of GBV as a form of the social norm which cannot be questioned (Piedalue et al., 2020). However, these claims are supported by mere examples and small-scale interview-based data. Thus, the research community lags a data-driven research that justifies the claim with sufficient empirical results. In this research, we do a large-scale analysis of social network content to find evidence of confluence between the culture of a country and GBV. Next, we present the role of social media content in bridging the gap of data for various research.

### Social media content

Social media has now become the new language of people, and this has generated a massive amount of data for various research. Social media data has removed the bottleneck of data requirements in numerous applications such as urban computing (Silva et al., 2019), cultural computing (Wang et al., 2017), personality computing (Samani et al., 2018) and many more. Social media content also plays a huge role in understanding people’s views and sentiments during the COVID-19 pandemic (Malagoli et al., 2021). Social media content has already substituted the tedious, time consuming, biased and under-represented survey-based data and has unlocked possibilities for research in many other directions. The utility of social media content increases with the availability of location-tagged data. The location-tagged online content has been used in numerous ethnographic research (Abdullah et al., 2015), cultural research (Cheke et al., 2020), and sociological research (Stewart et al., 2017) in recent days. Social media content is not mere data, but it captures several dimensions of human interests, psychology, and behavior. There have been works which found that the social media content reflects the real-world properties as well. García-Gavilanes, Mejova & Quercia (2014); Garcia-Gavilanes, Quercia & Jaimes (2013) have found that interaction and usage of social networks are dependent on social, economic, and cultural aspects of users. Thus, the real-world behavior of people is also mirrored in social networks. This utility of social media content motivates us to examine the relationship between GBV and a country’s culture through analysis of social media data. For this research, we use Twitter data related to GBV from different countries.

### Measuring culture

Culture is an amalgamation of thoughts, beliefs, potential acts, and a lot more. A number of definitions of culture are available from previous works (Hofstede, Hofstede & Minkov, 2005). One of many definitions of culture is *“a fuzzy set of assumptions and values, orientations to life, beliefs, policies, procedures and behavioural conventions that are shared by a group of people”* (Spencer-Oatey & Franklin, 2012). Culture plays a vital role in many spheres of life, such as behaviour (Huang, Beshai & Yu, 2016) economy (Herrero, Jiménez & Alcalde, 2021), language (Sazzed, 2021), attitude (Shin, Chotiyaputta & Zaid, 2022). In order to ease out research based on culture, there have been several quantification of culture. Hofstede has done one such comprehensive quantification. Hofstede (Hofstede, Hofstede & Minkov, 2005) defines culture in terms of six parameters (*Power Distance, Uncertainty Avoidance, Individualism, Masculinity, Long Term Orientation, Indulgence*) and quantifies each one for different countries of the world. An extensive set of previous works use Hofstede Dimensions to quantify culture (García-Gavilanes, Mejova & Quercia, 2014; Garcia-Gavilanes, Quercia & Jaimes, 2013; Prakash & Majumdar, 2021). For this research, we also used Hofstede’s dimensions which have been used in a huge number of research to measure culture.

### GBV through the lens of social media

Social media provides an uncensored and user-friendly medium for expressing views and opinions (Puente, Maceiras & Romero, 2021). With this, social media has become a platform for self-expression as well as for conducting online campaigns (Martínez, Pacheco & Galicia, 2021). There have been many campaigns on social media related to GBV like the #metoo, #Notokay, #StateOfWomen, #HeForShe and many more (Karuna et al., 2016). These campaigns and freedom of expression on social media have generated huge data related to GBV. The recent campaign of #Metoo observed an unprecedented response from all around the globe, thus, generating huge data related to GBV. And the event was followed by a sudden upsurge in research related to GBV using the generated data.

Thus, the data availability of social media has helped in many recent works related to GBV, which have delivered a multitude of interesting findings (Moitra, Ahmed & Chandra, 2021; Razi, 2020; Pandey et al., 2018; Khatua, Cambria & Khatua, 2018). Moreover, location-tagged social media data also assist in several cross-cultural studies related to GBV (Purohit et al., 2015; Starkey et al., 2019). In this article, we also used social media Twitter data from different countries of the world as a source of data.

## Methodology

**Figure 1:**
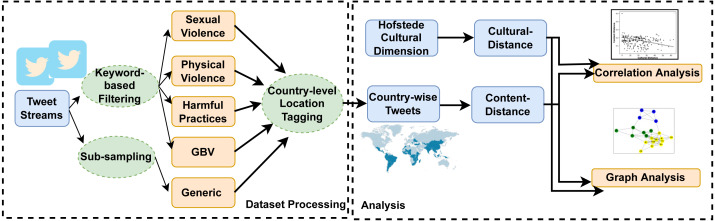
An overview of our proposed methodology.

In this section, we first give details of the collected dataset and its processing. Further, in the section, we elaborate on the methodology used to understand the relation between GBV and culture. We process the country-level tweets from different categories for correlation analysis and graph analysis w.r.t culture of different countries. The flow of methodology is represented in Fig. 1. Next, we explain the details of data collection and its processing to obtain country-wise GBV tweets.

### Dataset and processing

We use public streams of Twitter data collected using the Twitter Streaming API. We procured 1% of public tweets provided by the API for a period of two years and five months (1st July 2016–25th Nov 2018). We remove all the duplicate tweets and retweets from the collected data as these do not add any new information (Cheke et al., 2020). From the collected tweets, we extracted GBV related tweets using a keyword matching approach as described next.

### GBV Tweet extraction

UNFPA (United Nations Population Fund) domain experts have proposed three categories of GBV, namely *sexual violence*, *physical violence*, and *harmful practices*. They have also provided unique keywords related to each category of GBV, which have been used frequently in past literature for GBV related research (Purohit et al., 2015; ElSherief, Belding & Nguyen, 2017). Table 1 shows a total of 81 keywords constituting 29, 25, and 27 keywords from sexual violence, physical violence, and harmful practices respectively. We use the same keywords to extract relevant tweets from all three categories. The keyword set provided by UNFPA is very precise and can contain multi-words. Our methodology for extracting tweets for a particular keyword is based on the presence of the keyword in a tweet. If all the words of a multi-word keyword are present in a tweet regardless of order, we consider it a match. For example, for the category sexual violence, *sexual assault* is a related keyword with two words. If a tweet contains both the words *sexual* and *assault*, we consider it a match. For the cases where a tweet matches more than one category, we consider the tweet in both categories of GBV. This approach has been used in previous works in order to deliver high-precision data (Purohit et al., 2015). From the keywords related to each category of GBV, we extract tweets and create a tweet dataset from three categories, namely the sexual violence dataset, physical violence dataset, and harmful practices dataset, with a total of 0.83*million*, 0.53*million*, and 0.66*million* tweets, respectively. Further, we combined all three category tweets to create a GBV tweet dataset containing more than two million tweets.

**Table 1:** Set of keywords to identify tweets from different categories.

Category	Relevant keywords
Sexual violence	sexual assault, sexual violence, woman/women/girl/female harass, woman/women/girl/female attacked, boyfriend/boy-friend assault, stalking woman/women/girl/female, groping woman/women/girl/female, sexual/rape victim, gang rape, victim blame, sex predator, woman/women/girl/female forced
Physical violence	woman/women/girl/female beat up, woman/women/girl/female acid attack, woman/women/girl/female violence, woman/women/girl/female punched, woman/women/girl/female attacked, gender/domestic violence, intimate partner violence, physical abuse/violence
Harmful practices	child/children/underage/forced marriage, sex/child/children trafficking, woman/women/girl/female trafficking, child molestation/bride/sex, child violence/abuse/bullying/beat, spouse abuse, sex/women/forced slave, female genital mutilation (fgm), early marriage, pedophilia, human trafficking, woman abuse
GBV	All the keywords from sexual violence, physical violence, and harmful practices

### Generic Tweet dataset

We created another dataset, the *generic tweet dataset*, to provide a better context of comparison with other categories of the dataset. This dataset is used for drawing inferences from GBV categories dataset w.r.t a generic dataset. For creating this dataset, we borrowed the methodology of ElSherief, Belding & Nguyen (2017). Our collected data is for a very long period, resulting in around 4 billion tweets. We extracted a random 1% sub-sample of total collected tweets as a *generic tweet dataset*. To eliminate duplicate content here as well, we removed tweets which are duplicates and retweets. Details of generic tweets data are given in Table 2.

**Table 2:** Descriptive statistics of collected data.

Data category	Before location tagging	After location tagging
Sexual violence	836,497	681,537
Physical violence	534,707	433,560
Harmful practices	659,666	522,844
GBV tweets	2,030,874	1,637,941
Generic tweets	42,445,234	36,689,133

### Country-level location tagging

There are many indicators of location in a tweet, such as *geotags*, *time zone*, and *profile location*. Adopting the location indicators of Annamoradnejad et al. (2019) for tagging each tweet to a location, we use a three-level hierarchy of location indicative according to their accuracy levels (Kulshrestha et al., 2012). The first one is geotag, which gives the most accurate location information. If a geotag is available, then we use it for location tagging, and if it is not present, we look for the time zone data. Time zone is also an accurate way to tag country-level locations. A time zone data directly contains the user’s time zone in the form of the corresponding country name. For the cases where even time zone information is not available, then we look for the next location information in the hierarchy, *i.e.,* location field mentioned in the user profile. Geotags and time zone contain exact country names, which can be directly mapped to a country. User profile location is an unstructured text location field that requires further processing to get country information. For this, we use the approach used by Dredze, Osborne & Kambadur (2016) where city names present in the user profile location are mapped to corresponding country names based on the Geoname (https://pypi.org/project/geonames/) world gazetteer. We borrow the list of required countries from Annamoradnejad et al. (2019) where authors have used a list of 22 countries, namely *Arab countries, Argentina, Australia, Brazil, Canada, China, Colombia, France, Germany, India, Indonesia, Iran, Italy, Japan, Korea, Philippines, Russia, Spain, Thailand, Turkey, UK (United Kingdom), USA(United States of America)*. *Arab Countries* is a group of countries with a similar culture, so we merged tweets from all *Arab Countries*. There were very few tweets from Korea (170), so we discarded Korea from the list of considered countries and limited our research to the remaining 21 countries, each having more than 3,000 tweets. We apply the same location tagging scheme to all the GBV tweets and generic tweets. The complete data statistics are shown in Table 2 for all the categories of tweets.

We present the evaluation methodology and evaluation results of GBV tweets extraction and location tagging in ‘Results’. In this research, we want to explore the relationship between GBV online content and culture of a country. For this, we perform two analyses i. Correlation Analyses and ii. Graph Analyses. In correlation analysis, we correlate culture and its dimensions with different categories of online content in order to understand their relationship. In graph analyses, we create country graphs on the basis of parameters correlated in correlation analyses like content, and culture, which are compared using multiple graph comparison metrics in order to re-assure the observed relationships from correlation analyses. Next, we discuss the methodology used for these analyses.

### Correlation analysis

In order to find a relation between the culture of a country and GBV, we calculate cultural distance and content distance between each pair of countries as detailed next.

### Cultural distance

We quantify the cultural distance between two countries using cultural dimensions proposed by Geert Hofstede (Hofstede, Hofstede & Minkov, 2005). Geert Hofstede administered a huge survey among people from different countries to measure the difference in the way they behave. He has quantified six dimensions of culture (*power distance*^1^, 1This is a measure of the level of acceptance of unequal power in society.*individualism*^2^, 2This is a measure of rights and concerns of each person rather than for a group or community.*masculinity*^3^, 3This is a measure of the distribution of gender-based roles in society.*uncertainty avoidance*^4^, 4This is a measure of likeliness that people avoid uncertainty.*long-term orientation*^5^, 5This parameter measures the characteristics of perseverance and futuristic mindset among people.*indulgence*^6^) 6This measures the degree of fun and enjoyment a society allows.for different countries in values ranging between 0–120. In order to measure cultural distance between two countries, we adopt the formulation of (Annamoradnejad et al., 2019) where authors use the euclidean distance between two countries to measure the cultural distance. The cultural distance can be formulated as shown in Eq. (1), where |*D*| is the total number of dimensions, ${d}_{{c}_{1}}^{i}$ and ${d}_{{c}_{2}}^{i}$ are the values of dimension *d*^*i*^ for countries *c*_1_, *c*_2_ respectively.

(1) \begin{eqnarray*}Cultural~Distance({C}_{1},{C}_{2})=\sqrt{\sum _{i=1}^{{|}D{|}}({d}_{{c}_{1}}^{i}-{d}_{{c}_{2}}^{i})^{2}}\end{eqnarray*}

We also calculate the distance between countries on the basis of each dimension of culture proposed by Hofstede. For example, *power distance* is one of the dimensions of culture, and we need to calculate the distance between two countries according to *power distance*. For this also, we use euclidean distance, but since there is only one parameter, this becomes equivalent to |*d*_*c*_1__ − *d*_*c*_2__|. For further analyses, we calculate the cultural distance for each pair of countries on the basis of culture and six dimensions of culture.

### Content distance

Online content related to a particular topic from a particular country captures country-level user comments and discussions on that topic (Cheke et al., 2020). In order to measure the difference between contents from two countries, we measure the *content distance* between two countries using Jaccard Similarity. First, all the tweets from each country are pre-processed to generate country-wise tweet tokens, details of which are given next.

### Tweet preprocessing

We adopt the pre-processing settings of Cheke et al. (2020) to generate tweet tokens from each country. We first remove URLs, mentions, punctuation, extra spaces, stop words, and emoticons. Online acronyms and short forms are expanded using NetLingo (https://www.netlingo.com/acronyms.php). For hashtags, we removed the symbol # and kept the remaining word. Spelling and typos are corrected using Textblob (https://textblob.readthedocs.io/en/dev/). We also transliterated non-English words to English to reduce inconsistencies in language. Lastly, we tokenized each tweet using NLTK (Natural Language Toolkit). Extracted tokens from all the tweets of a country are merged to create country-wise tweet tokens. Next, for each pair of countries, we calculate *content distance* using the formula shown in Eq. (2), where *C*_1_, *C*_2_ are the set of all the tweet tokens of countries *C*_1_ and *C*_2_, respectively, and $ \frac{{|}{C}_{1}\cap {C}_{2}{|}}{{|}{C}_{1}\cup {C}_{2}{|}} $ is the Jaccard Similarity.^7^ 7Jaccard similarity is a popular metric for measuring content similarity. We also experimented with Cosine similarity, another popular metric and found similar result patterns.

(2) \begin{eqnarray*}ContentDistance({C}_{1},{C}_{2})=1- \frac{{|}{C}_{1}\cap {C}_{2}{|}}{{|}{C}_{1}\cup {C}_{2}{|}} .\end{eqnarray*}

We have a total of 5 categories of online content *i.e.,* sexual violence, physical violence, harmful practices, GBV, and generic content. We apply the same methodology to extract country-wise tweet tokens from each category of online content.

### Correlation

A correlation helps in understanding the relationship between two variables. *Pearson correlation* and *Spearman correlation* are two popular metrics for correlation. To establish robustness in our findings, we use both *Pearson correlation*, and *Spearman correlation* for calculating the association between *content distance* and *cultural distance*. *Pearson correlation* captures the linear relationship between two variables and *Spearman correlation* captures the monotonic relationship between two variables. Both the correlation metrics give correlation values in the range of (−1 to +1). A positive correlation value indicates that content similarity is higher for countries having higher cultural similarity and a negative correlation indicates vice versa. For calculating the correlation, we calculated content distance and corresponding cultural distance for each country pair (a total of ^*n*^*C*_2_ pairs, if there are *n* countries). For exhaustive correlation analysis, we measured multiple correlations by keeping one correlation variable as different types of content (sexual, physical, harmful, GBV, and generic tweets) and another variable as six dimensions of culture.

### P-value

For measuring the fitness of a correlation, we calculated the *p*-values for each correlation using the python library SciPy (https://docs.scipy.org/doc/scipy-0.14.0/reference/stats.html). A *p*-value represents the measure of occurrence of the correlation between two data samples by chance.

### Graph analysis

Our objective is to compare GBV related content to a country’s culture. To this end, we created country graphs where edge weights are decided on the basis of different distances in terms of GBV content and culture, as mentioned in ‘Correlation Analysis’. For detailed analyses, we created multiple weighted graphs among countries with a different edge parameters. Finally, we compared created graphs using multiple graph distance metrics and graph clustering.

**Country graph**: A country graph created in this research is an un-directed, weighted graph *G* = (*C*, *E*), where *C* denotes the nodes of the graph, which are countries, and *E* denotes the set of edges between countries. For all the graphs in this research, the set of nodes *C* and the set of edges *E* are the same. The only difference is in the weights of the edges. Next, we describe the creation of edges in the required graphs.

**GBV content graph**: This graph captures the relationship between countries according to GBV content distance. In GBV content graph *G*_*gbv*_ = (*C*, *E*), the weights of the set of edges *E* are decided on the basis of the *content distance* score between two countries on the basis of GBV tweets. Here, GBV tweets are used for calculating content distance. We also create *sexual violence graph*, *physical violence graph*, and *harmful practices graph* where for assigning edge weights, we calculate content distance on sexual violence tweets, physical violence tweets, and harmful practices tweets, respectively.

**Generic content Graph**: This graph captures the relationship between countries and generic content. In the generic content graph *G*_*rand*_ = (*C*, *E*), the weights of the edges are assigned according to the *content distance* score between two countries on the basis of generic tweet data.

**Cultural graph**: This graph captures the cultural relationship between countries. In the cultural graph *G*_*cult*_ = (*C*, *E*), the weights of the edges are decided by the value of cultural distance calculated using Eq. (1).

**Graph Pre-processing**: For all the graphs *G* = (*C*, *E*), there is an edge between any pair of countries with a weight creating a complete graph. Further, all the created graphs have a different range of values of edge weight. For example, for GBV tweets graphs, edge weights will lie in the range (0,1), but for the culture graph, the values of weights can range from (0–120). To ensure consistency, we upscale edge weights in the range of (0,1) to a range of (0–120). Next, we pruned edges that are unimportant, *i.e.,* whose weight is lesser than the median of all the edge weights. Thus, keeping only the important, *i.e.,* higher edge weight edges in the graph. Before pre-processing, each graph is a complete graph with the same edges in all the graphs, but after pre-processing, each graph is a non-complete graph with only important edges resulting in different edges in each graph. The same is applied to all the graphs, and the final pre-processed graph is a weighted, un-directed, and non-complete graph.

We also mention that all the distances (content/culture) used to decide edge weight in all the graphs follow the axioms of distance (Kosub, 2019).

#### Graph comparison metrics

For comparing two graphs, past literature has proposed a number of metrics depending upon the type of graphs (Tantardini et al., 2019; McCabe et al., 2020). In this research, we have graphs with node correspondence, *i.e.,* same nodes in every graph. Additionally, our graphs are un-directed and weighted. For the purpose of graph comparison, we use multiple graph distance metrics to calculate the distance between two graphs. Graph distance shows how dissimilar the two graphs are. For calculating graph distance, we used the Python library *netrd* (https://netrd.readthedocs.io/en/latest/distance.html). Next, we describe metrics used in our research to calculate graph distance.

### Distances:

• Quantum JSD: Quantum Jensen–Shannon Divergence (De Domenico & Biamonte, 2016) compares two weighted and undirected graphs by finding the distance between spectral entropy of density matrices.
• Degree divergence: This method (Hébert-Dufresne, Grochow & Allard, 2016) compares the degree distribution of two graphs. This methodology is applicable to weighted as well as unweighted graphs but only undirected graphs.
• Jaccard distance: Jaccard distance (Oggier & Datta, 2021) is applicable to only unweighted graphs, and its value depends on the number of common edges in the two compared graphs. For applying to our graphs, we coerced weighted graphs into unweighted ones by removing weights from all the graphs.
• Hamming distance: Hamming distance is one of the popular techniques for measuring the distance between two unweighted graphs. This is a measure of element-wise disagreement between the two adjacency matrices of the graphs. We applied Hamming distance to our graphs by coercing weighted graphs to unweighted ones by simply removing the weights.
• HammingIpsenMikhailov: This method is an enhanced version of Hamming Distance which takes into account the disagreement between adjacency matrices and associated laplacian matrices. This is applicable to weighted and undirected graphs.
• Frobenius: This is an adjacency matrix level distance that calculates L2-norms of the adjacency matrices.
• NetLSD: A metric for measuring graph distance based on spectral node signature distributions for unweighted graphs. For this, we coerced our graphs to unweighted ones by removing the weights.

#### Graph clustering

A graph clustering algorithm clusters similar nodes in different groups. If two graphs are similar, then their clusters will also be similar. In order to compare the GBV graph and the generic graph with the culture graph, we used the Louvain community detection algorithm (De Meo et al., 2011). Louvain community detection algorithm is a clustering algorithm for nodes of a weighted graph where nodes are clustered on the basis of modularity between the nodes. Here the number of clusters was decided by the algorithm only.

## Results

In this section, we first provide validation results for our proposed methodology of GBV tweet filtering and location tagging. Then we present the results and insights of correlation analyses and graph analyses in order to understand the correspondence between GBV and culture.

### GBV tweet extraction and error analysis

GBV tweet extraction is accomplished by tagging tweets using a keyword matching process. Following the keyword match verification methodology of Cheke et al. (2020), we employed three graduate annotators to manually tag the GBV category. Annotators were provided with a sample of tweets without any category information and were asked to manually tag each tweet to one or more categories of GBV (sexual violence, physical violence, harmful practices) with their own understanding and external online resources. Annotators were provided with a basic definition of GBV and its categories. For the purpose of validation, we created a balanced and shuffled sample of 6,000 tweets with 2,000 tweets from each category of GBV. For each tweet annotated by the three annotators, we select the majority category as the final category. Tweets that do not have any majority category are discarded. Considering the category tagged by annotators as the actual categories, we calculate the precision value of our keyword matching methodology for each category of GBV. The precision value for sexual violence is found to be 0.97, for physical violence 0.96, and for harmful practices to be 0.98.

Table 3 shows a few example tweets and tagged GBV categories. Examples 1–9 shows matching keywords and the tagged GBV category of the tweets from all three categories of GBV. Example 10–11 show tweets that contain keywords from more than a category of GBV. These tweets are kept in all the matching categories. In examples 12–13, keyword matching results in the wrong tagging of tweets because of contextual differences in tweets. As we can see in example tweet 12, the keywords *woman* and *attacked* belong to physical violence keywords, and hence the tweet is wrongly classified in the physical violence category. There are only a few such errors in GBV tweet category tagging arising because of changes in the context of tweets.

**Table 3:** Example tweets with their matching keywords and tagged GBV category.

S.No.	Example tweets	Category
1	Why is **harassment** an automatic career hazard for a **woman** receiving any amount of professional attention?	Sexual violence
2	**Girls** are **forced** to sleep and authorities are POWERLESS. Europe is dead.	Sexual violence
3	Yesterday I asked my daughter’s schools to stop **slut-shaming** and **victim-blaming** girls. It went viral.	Sexual violence
4	#Berlin metro attacker who kicked **woman** down stairs in random act of **violence** detained	Physical violence
5	laws don’t cause divorce, **domestic violence** does	Physical violence
6	Some **girls** are **beaten up** by their boyfriends and stick around saying Ï see something in him.	Physical violence
7	Gather round children, I’m doing a thread on how this society sexualizes **underage girls**. Leggo.	Harmful practices
8	If you suspect a **child** is being **abused** You have a moral duty to report it	Harmful practices
9	A 12 year old **child bride** taking photos in her wedding dress. Can you imagine it?	Harmful practices
10	For most of these **women**, a history of **sexual abuse** and **childhood** trauma dragged them into prostitution	Multiple
11	She has written books on **sexual abuse**, **child molestation**, **domestic violence**.	Multiple
12	**Woman** With Too Much Makeup Mistaken As Clown; **Attacked** By Angry Mob	Physical (Error)
13	all I want for my children is happiness! I don’t care what their **sexual** preference Is.. No one can **force** them	Sexual (Error)

### Evaluation of location tagging

We have a three-level hierarchy(time zone, geotags, profile location) of location tagging. Location tagging from time zone and geotags is completely accurate. For evaluating location tagging from profile location a random sample of 10, 000 tweets are given to three independent graduate annotators who were asked to manually tag a country-level location from their own understanding using online gazetteers and searches. The majority country name is selected as the final tagged country name. The profile location field with no majority among annotators is discarded. Considering the country tagged by manual annotation as the actual country, we obtained a precision score of 0.94.

### Correlation analysis

Tables 4 and 5 show the results of *pearson correlation* and *spearman correlation* of different types of online content with culture and its parameters. From the tables, we can draw the following observations.

**Table 4:** Pearson correlation coefficient and *p*-values between different cultural distances and different content distances. All the correlations all calculated on a sample of 210 country pairs, since there are 21 countries. Degree of freedom for the correlation analyses is 208.

	GBV tweets		Sexual violence		Physical violence		Harmful practices		Generic tweets	
	Corr	*p*-value	Corr	*p*-value	Corr	*p*-value	Corr	*p*-value	Corr	*p*-value
Cultural distance	0.55	0.001	0.51	0.001	0.53	0.001	0.53	0.001	0.33	0.001
Power distance	0.27	0.001	0.30	0.001	0.30	0.001	0.32	0.001	0.12	0.01
Masculinity	0.16	0.001	0.16	0.001	0.18	0.001	0.16	0.001	0.17	0.01
Uncertainty avoidance	0.36	0.001	0.34	0.001	0.31	0.001	0.33	0.001	0.23	0.001
Long-term orientation	0.17	0.001	0.16	0.001	0.19	0.001	0.18	0.001	0.21	0.001
Indulgence	0.34	0.001	0.30	0.001	0.32	0.001	0.30	0.001	0.13	0.01
Individualism	0.45	0.001	0.40	0.001	0.42	0.001	0.42	0.001	0.15	0.001

**Table 5:** Spearman correlation coefficient and *p*-values between different cultural distances and different content distances. All the correlations all calculated on a sample of 210 country pairs, since there are 21 countries. Degree of freedom for the correlation analyses is 208.

	GBV tweets		Sexual violence		Physical violence		Harmful practices		Generic tweets	
	Corr	*p*-value	Corr	*p*-value	Corr	*p*-value	Corr	*p*-value	Corr	*p*-value
Cultural distance	0.28	0.001	0.24	0.001	0.25	0.001	0.28	0.001	0.03	0.43
Power distance	0.17	0.001	0.23	0.001	0.21	0.001	0.25	0.001	0.01	0.82
Masculinity	0.07	0.11	0.07	0.13	0.07	0.11	0.05	0.26	0.06	0.14
Uncertainty avoidance	0.27	0.001	0.28	0.001	0.22	0.001	0.27	0.001	0.09	0.03
Long-term orientation	0.08	0.001	0.04	0.38	0.07	0.11	0.07	0.11	0.12	0.01
Indulgence	0.30	0.001	0.26	0.001	0.27	0.001	0.26	0.001	0.01	0.74
Individualism	0.35	0.001	0.33	0.001	0.36	0.001	0.37	0.001	0.02	0.73

1. GBV content and all categories of GBV content show a positive correlation with culture and all its parameters by both the correlation metrics. For example, the correlation between GBV content and culture is 0.55, with a significant *p*-value of 0.001. Similarly, the correlation between culture and other categories of GBV *i.e.,* sexual violence, physical violence, and harmful practices content is 0.51, 0.53, and 0.53, respectively with significant *p*-values.
2. The three parameters of culture *uncertainty avoidance*, *indulgence*, and *individualism* show comparatively higher correlation values as compared to other parameters of culture *power distance*, *masculinity*, and *long-term orientation*. This observation is consistent with all the categories of GBV content and with both the correlation metrics. The Pearson correlation of *uncertainty avoidance*, *indulgence*, and *individualism* with GBV tweet content is 0.36, 0.34, and 0.45 (Table 1). On the other hand, the pearson correlation of *power distance*, *masculinity*, and *long-term orientation* with GBV tweets content is 0.27, 0.16, and 0.17 respectively.
3. We also observe that the same content analyses performed for GBV content did not show a similar strong and consistent correlation with generic content. The Pearson correlation between generic content and culture is 0.33, which is much lower than the Pearson correlation between GBV content and culture, *i.e.,* 0.55. Additionally, generic content fails to show any correlation with culture and its parameters from spearman correlation.

**Figure 2:**
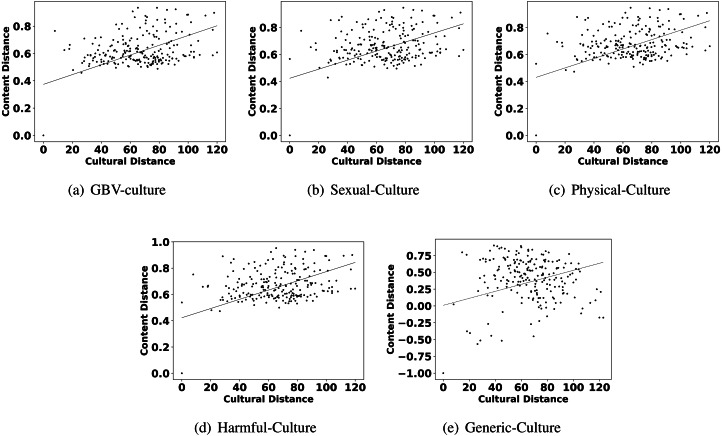
Scatter plots between different categories of content distance (A: GBV, B: sexual, C: physical, D: harmful practices, E: generic) and cultural distance.

**Figure 3:**
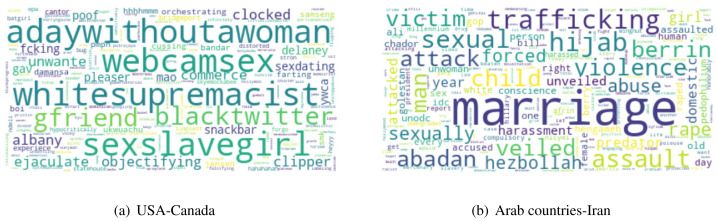
Word Clouds showing common frequent keywords of culturally similar countries (A: USA-Canada and B: Arab Countries-Iran).

Observation 1 indicates that GBV content has an influence of culture and all six parameters of culture. The observation is consistent with all the categories of GBV content, *i.e.,* sexual violence, physical violence, and harmful practices. Additionally, we also show the scatter plots of the culture and different GBV content types in Fig. 2 to reconfirm the findings. All these results imply that countries with similar culture also show higher similarity in GBV content. GBV content is composed of discussions, news, comments generated by users on the topic related to GBV. The reason for the similarity in GBV content for similar culture countries is the similarity in their discussions, news, and comments. For further understanding, we manually discerned the content of similar culture countries. According to Hofstede’s dimensions, the USA and Canada are more similar^8^ 8This similarity is according to the Hofstede’s dimensions calculated using Eq. (1)in culture as compared to USA and Iran. Similarly, Iran is more similar to Arab countries as compared to the USA. Table 6 shows the scores of cultural distance between different countries using Hofstede’s dimensions. In order to show the content differences of different culture countries, we ploted the word clouds of common frequent words of USA-Canada and Arab countries-Iran in Figs. 3A and 3B, respectively. For the countries USA and Canada, we found keywords like *gfriend, whitesupremacist, objectifying* as common frequent keywords. For the countries Arab countries and Iran, we found keywords like *veiled, hijab, attacked, predator* as common frequent keywords.

**Table 6:** Cultural distance between different countries by Hofstede’s dimensions.

	Arab Countries	Argentina	Australia	Brazil	Canada	China	Colombia	France	Germany	India	Indonesia	Iran	Italy	Japan	Philippines	Russia	Spain	Thailand	Turkey	UK	USA
Arab Countries	0	46.3	78.9	35.6	72.1	78.7	59.7	59	81.7	41.7	50.6	28.3	64.7	85.8	31.8	69.2	42.9	34	35.9	89.1	78.4
Argentina	46.3	0	58.3	34.2	54.8	104	44.9	56.6	74.4	71.5	75.6	38.9	62.7	80.9	67	89.1	36.9	47.7	35.9	75.8	61.7
Australia	78.9	58.3	0	71.4	20.5	117	88.4	71.8	74.4	79.8	102	64.9	66.1	101	86.4	120	70.3	85.3	78.1	34.5	7.9
Brazil	35.6	34.2	71.4	0	60	77.5	48.8	41.7	65.4	51.5	47.3	41.5	58.5	70.1	49.7	63.7	26.8	32.6	14.5	76.7	71.6
Canada	72.1	54.8	20.5	60	0	102	84.8	60.2	60.4	67.4	86.7	58	57.7	92.5	79.1	107	58.8	73.2	66.8	26.3	18.2
China	78.7	104	117	77.5	102	0	108	87	75.9	48.3	40.1	89.6	82.4	79.8	66.9	75.5	84.7	77.4	79.7	101	112
Colombia	59.7	44.9	88.4	48.8	84.8	108	0	87.3	104	87.5	76.9	59.7	97.5	98.3	65.4	104	69.4	54.8	56.3	102	91.4
France	59	56.6	71.8	41.7	60.2	87	87.3	0	50.1	59.3	70	65.4	39.1	64.8	75.7	53.6	28.1	64.8	38.6	71.9	70.6
Germany	81.7	74.4	74.4	65.4	60.4	75.9	104	50.1	0	63.9	76.1	81	31.5	49	90.7	79.8	54.9	81.9	64.6	56.9	70.8
India	41.7	71.5	79.8	51.5	67.4	48.3	87.5	59.3	63.9	0	39.7	50.3	55.3	79.8	37.7	68.8	55.1	52.3	54.3	73.8	75.4
Indonesia	50.6	75.6	102	47.3	86.7	40.1	76.9	70	76.1	39.7	0	60	77.4	81.4	46.1	62.1	59.2	40	49.4	95.7	99.3
Iran	28.3	38.9	64.9	41.5	58	89.6	59.7	65.4	81	50.3	60	0	68.4	96.7	47.3	87.1	44.7	30.6	43.1	78.7	65.3
Italy	64.7	62.7	66.1	58.5	57.7	82.4	97.5	39.1	31.5	55.3	77.4	68.4	0	51.7	78.6	72.6	44.3	76.6	56	60.8	63.1
Japan	85.8	80.9	101	70.1	92.5	79.8	98.3	64.8	49	79.8	81.4	96.7	51.7	0	93.4	74.7	67.1	91.9	68	91.8	100
Philippines	31.8	67	86.4	49.7	79.1	66.9	65.4	75.7	90.7	37.7	46.1	47.3	78.6	93.4	0	82.6	66.2	48.7	56.8	90.2	84.2
Russia	69.2	89.1	120	63.7	107	75.5	104	53.6	79.8	68.8	62.1	87.1	72.6	74.7	82.6	0	57.1	72.6	55.2	117	118
Spain	42.9	36.9	70.3	26.8	58.8	84.7	69.4	28.1	54.9	55.1	59.2	44.7	44.3	67.1	66.2	57.1	0	42.6	17.9	76.1	70.5
Thailand	34	47.7	85.3	32.6	73.2	77.4	54.8	64.8	81.9	52.3	40	30.6	76.6	91.9	48.7	72.6	42.6	0	32.6	91.8	85.4
Turkey	35.9	35.9	78.1	14.5	66.8	79.7	56.3	38.6	64.6	54.3	49.4	43.1	56	68	56.8	55.2	17.9	32.6	0	84	78.5
UK	89.1	75.8	34.5	76.7	26.3	101	102	71.9	56.9	73.8	95.7	78.7	60.8	91.8	90.2	117	76.1	91.8	84	0	28.5
USA	78.4	61.7	7.9	71.6	18.2	112	91.4	70.6	70.8	75.4	99.3	65.3	63.1	100	84.2	118	70.5	85.4	78.5	28.5	0

**Figure 4:**
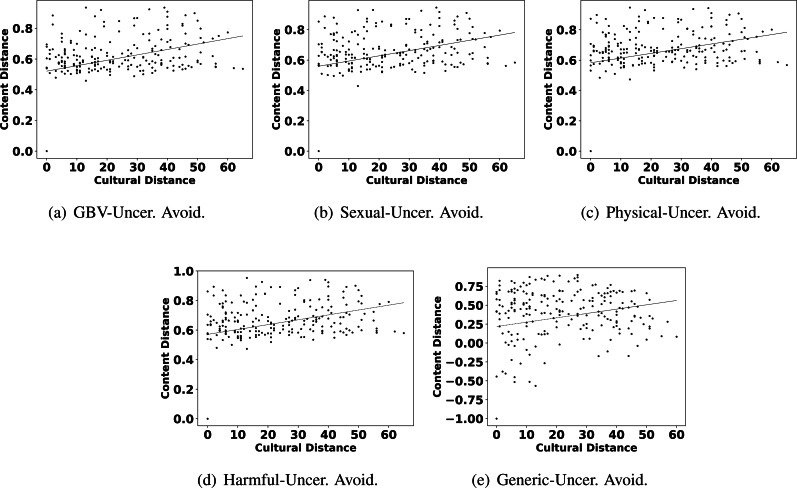
Scatter plots between different categories of content distance (A: GBV, B: sexual, C: physical, D: harmful practices, E: generic) and cultural distance (uncertainty avoidance).

**Figure 5:**
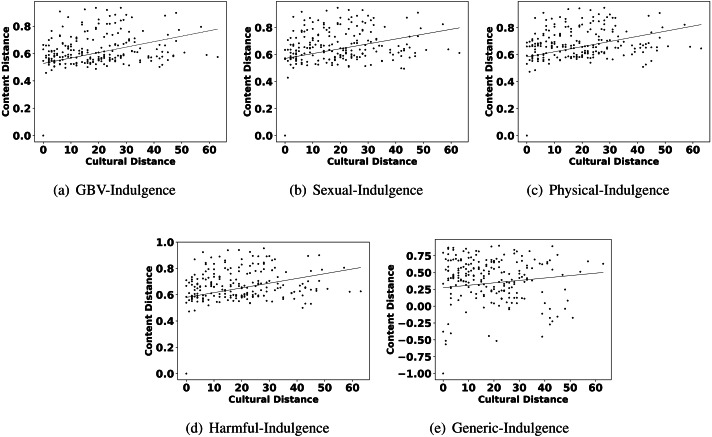
Scatter plots between different categories of content distance (A: GBV, B: sexual, C: physical, D: harmful practices, E: generic) and cultural distance (indulgence).

**Figure 6:**
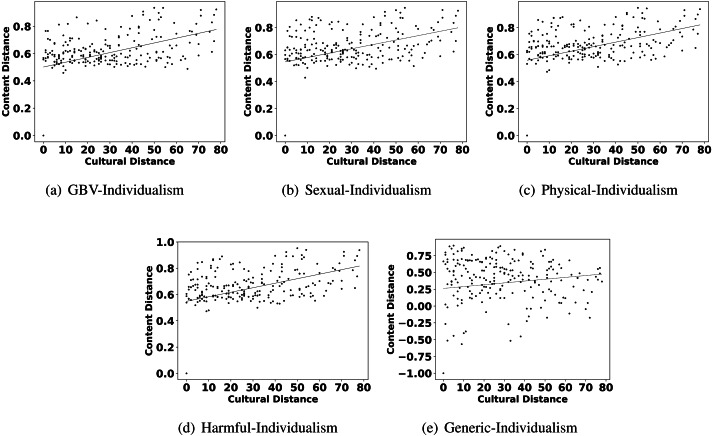
Scatter plots between different categories of content distance (A: GBV, B: sexual, C: physical, D: harmful practices, E: generic) and cultural distance (individualism).

The highlights in observation 2 suggest that a few parameters of culture also play an important role in shaping the content related to GBV. Interestingly, Hofstede’s parameters uncertainty avoidance, indulgence, and individualism are found to show more impact on GBV related content than other parameters like power distance, masculinity, and long term orientation. For further reconfirming the connection between these parameters and different types of GBV content, we also show the scatter plots of these parameters and different content in Figs. 4, 5 and 6. From the scatter plots, we can evidently observe that Hofstede’s parameters uncertainty avoidance, indulgence, and individualism consistently show a close association (points are closer to the fitted line) with all types of GBV content. The same is not true for generic content. This shows a connection between these parameters of culture and different GBV categories content. The reasons for more influence of these parameters require further exploration which is outside the scope of this work. However, this observation again recommends a role of culture on GBV.

Observation 3 further strengthens the findings of Observations 1 and 2. The pattern of correlation showing a connection between culture and different categories of GBV is not the same for generic content. The lower and inconsistent correlation values of the generic content as compared to GBV content reinforce a stronger relationship between GBV content and the culture of a country. Further, the scatter plots shown in Figs. 2, 4, 5 and 6 also show that the points in all the plots of generic content are more scattered from the fitted line as compared to points in GBV and its category content plots. For example, the points in the GBV-culture plot (Fig. 2A) are closer to the fitted line, while in the generic-culture plot (Fig. 2E), the points are farther to the fitted lines showing a comparatively lower correlation. Other categories of content (sexual, physical, and harmful) also show a stronger correlation with culture as compared to generic content.

Generic content is composed of content from different topics, a few of which can be highly correlated to culture (Cheke et al., 2020), such as food, and a few can hardly show any correlation (Annamoradnejad et al., 2019), such as technology. These characteristics of generic content can be the most probable reason for showing weak correlations. Here we showed results of generic content just to provide a broader background for understanding. Next, we describe the results from graph analyses in order to validate findings from correlation analyses.

### Graph analysis

We first summarize the statistical characteristics of the created graphs in our research in Table 7. All the graphs show similar basic properties because there is node correspondence in all the graphs. The edges and edge weights are the main varying parameter in the graphs. Next, we explain the details of graph comparison.

**Table 7:** Statistical inferences from the created graphs.

Graph attributes	GBV	Culture	Sexual	Physical	Harmful	Generic
Edges	105	105	105	105	105	105
Nodes	21	21	21	21	21	21
Clustering coefficient	0.71	0.74	0.73	0.69	0.71	0.80
No of nodes in largest connected component	21	21	20	20	19	19
No of connected components	1	1	2	2	3	3

Table 8 shows the distance between different created graphs from various metrics. For each distance metric, if the distance value between a pair of graphs (G1, G2) is smaller than the distance between another pair of graphs (G3, G2), it means that the graph G2 is more similar to G1 as compared to G3. From the table, we observe that for all the metrics, the distance between the generic tweet graph and the culture graph is consistently higher than the distance between other graphs (GBV-culture, sexual violence-culture, physical violence-culture, and harmful practices-culture). For example, the metric *QuantumJSD*, the distance between generic graph and culture graph is 0.27 while for GBV graph and culture graph is 0.21. For the same metric, the distance between the sexual graph-culture graph, the physical graph-culture graph, and the harmful graph-culture graph is 0.21, 0.20, and 0.22, respectively. This shows that the graph created using content distance by GBV and its categories are more similar among themselves and to the graph created using cultural distance. The graph created using the generic content is consistently more distant from the cultural graph as compared to other graphs. This observation re-validates the observation from correlation analyses showing a higher degree of similarity between GBV content for similar culture countries. As two similar graphs show lesser distance by graph distance metrics, two similar graphs will also show similar clusters. Next, we present clustering in the created graphs.

**Table 8:** Comparative results of distances between different graphs.

Distance metric	GBV-Culture	Sexual-Culture	Physical-Culture	Harmful-Culture	Generic-Culture
QuantumJSD	0.21	0.21	0.20	0.22	**0.27**
DegreeDivergence	0.19	0.26	0.34	0.37	**0.38**
JaccardDistance	0.70	0.70	0.72	0.71	**0.76**
Hamming	0.32	0.29	0.31	0.31	** 0.41**
HammingIpsenMikhailov	0.25	0.24	0.25	0.26	**0.32**
Frobenius	12.16	11.66	12.0	12.0	** 13.71**
NetLSD	2.71	11.62	11.18	11.97	**21.78**

### Clustering the graphs

**Figure 7:**
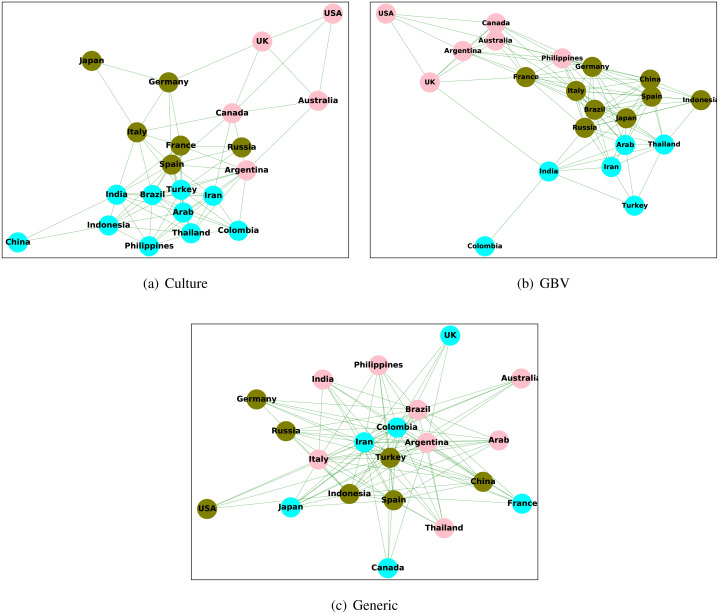
Clustering nodes (countries) in the culture graph, GBV graph, and generic graph.

Figure 7 shows the plots of clusters in the culture graph, GBV graph, and generic content graph. From the created clusters, we observe that the clusters in the culture graph are more similar to GBV graph as compared to generic graph. The countries in the culture graph which belong to the same cluster have a larger overlap with the GBV graph rather than the generic graph. For example, the countries *USA, UK, and Australia* belong to the same cluster in the culture graph, and the same is also true for GBV graph. However, for the generic graph, all three countries belong to different clusters. This observation again shows a higher similarity between the culture graph and the GBV graph than the generic graph and the culture graph. Thus, we observe that the created clusters are also congruous to all other findings stating a higher level of relation between culture and GBV content, which is not the same for generic content.

## Discussions

### Implications

In this research, we use social media data to verify connections between the culture of a country and GBV. Our findings suggest that real-world hypotheses are also evident in social media data, and their verification is no longer dependent on survey-based data. We believe that this research not only validates the hypothesis of confluence between culture and GBV but also points to the possibility of verification of other hypotheses related to GBV.

A finer analysis can also reveal culture-specific traits of GBV, which can further enhance understanding of GBV across cultures. We argue that these analyses are vital for designing culture-aware policies and strategies to curb GBV. There is a huge possibility of discovery of many more cultural norms like those pronounced by the World Bank (https://thedocs.worldbank.org/en/doc/656271571686555789-0090022019/original/ShiftingCulturalNormstoAddressGBV.pdf), which can promote GBV. Thus, this research paves a path for understanding culture-specific GBV using online social network data.

### Limitations and critiques

In this section, we show a few possible limitations and how our research overcomes those. In this article, we have performed cross-cultural research using online content from Twitter. Here, we limit our research to English tweets only pertaining to two reasons. First, the GBV keywords are in English, resulting in a collection of English GBV content. Second, English has become the new lingua franca on Twitter (Choudhary et al., 2018), which delivers sufficient tweets for this research.

GBV data collection is based upon GBV keywords provided by UNFPA, which is a global organization. The provided keywords can be incomplete and non-exhaustive. There might have scope for increasing these keywords; however, GBV is a sensitive issue, and extending keywords without the intervention of social experts may introduce errors. So, we limit this research to globally available keywords only.

Online content is much inflected by a flux of ongoing news and events, which can lead to differences in data patterns in certain time periods. However, our research is based upon data from a long temporal span which diffuses such temporal inflections (Grieve, Nini & Guo, 2018).

There can be many more ways to capture the distance between countries in terms of GBV, but we have limited this to content distance using two common metrics (cosine similarity and jaccard similarity). The content distance used in this research captures the basic difference between tweet tokens of the two countries. However, the same methodology can be easily adapted to other twitter features and metrics.

## Conclusion and Future Work

The article investigates evidence of the confluence between culture and GBV with the help of social media content. Social media content is explored by means of correlation analyses and graph-based analyses to find the traces of culture in GBV related social media content. In this research, we find a noteworthy influence of culture on GBV related content which is not apparent in generic content. The observation is consistent with different analyses and metrics. This research not only claims higher confluence between GBV and culture but also paves a path for effective policy-making and research related to GBV by means of social media content. Social media content captures behavioral aspects related to GBV, which can be used for other investigations related to GBV. As a future work of this research, we would like to understand the role of other factors like economy, unemployment,and crises in GBV. Moreover, this research is a global analysis of different countries of the world. We would also like to extend the research to a finer scale of states or counties within a country.

## Additional Information and Declarations

### Data Availability

The following information was supplied regarding data availability: The data and code are available at Zenodo: Rimjhim, & Sourav Dandapat. (2022). Is Gender Based Violence a Confluence of Culture? Let’s look for empirical evidence via Social Media. https://doi.org/10.5281/zenodo.6820256.
